# Evaluation of an mHealth App (DeStressify) on University Students’ Mental Health: Pilot Trial

**DOI:** 10.2196/mental.8324

**Published:** 2018-01-23

**Authors:** Rebecca Anne Lee, Mary Elizabeth Jung

**Affiliations:** ^1^ Faculty of Health and Social Development University of British Columbia Kelowna, BC Canada

**Keywords:** mHealth, mindfulness, mental health, students

## Abstract

**Background:**

One in five Canadians experience mental health issues with those in the age range of 15 to 24 years being most at risk of a mood disorder. University students have shown significantly higher rates of mental health problems than the general public. Current university support services are limited by factors such as available staff and finances, and social stigma has frequently been identified as an additional barrier that prevents students from accessing these resources. Mobile health (mHealth) apps are one form of alternative health support that is discrete and accessible to students, and although they are recognized as a promising alternative, there is limited research demonstrating their efficacy.

**Objective:**

The aim of this study was to evaluate a mindfulness-based app’s (“DeStressify”) efficacy on stress, anxiety, depressive symptomology, sleep behavior, work or class absenteeism, work or school productivity, and quality of life (QoL) among university students.

**Methods:**

Full-time undergraduate students at a Canadian university with smartphones and Internet access were recruited through in-class announcements and on-campus posters. Participants randomized into an experimental condition were given and instructed to use the DeStressify app 5 days a week for 4 weeks. Control condition participants were wait-listed. All participants completed pre- and postintervention Web-based surveys to self-assess stress, anxiety, depressive symptomatology, sleep quality, and health-related QoL.

**Results:**

A total of 206 responses were collected at baseline, with 163 participants completing the study (86 control, 77 experimental). Using DeStressify was shown to reduce trait anxiety (*P*=.01) and improve general health (*P*=.001), energy (*P*=.01), and emotional well-being (*P*=.01) in university students, and more participants in the experimental condition believed their productivity improved between baseline and postintervention measurements than the number of participants expected to believe so randomly by chance (*P*=.01). The app did not significantly improve stress, state anxiety, physical and social functioning, and role limitations because of physical or emotional health problems or pain (*P*>.05).

**Conclusions:**

Mindfulness-based apps may provide an effective alternative support for university students’ mental health. Universities and other institutions may benefit from promoting the use of DeStressify or other mindfulness-based mHealth apps among students who are interested in methods of anxiety management or mindfulness-based self-driven health support. Future steps include examining DeStressify and similar mHealth apps over a longer period and in university staff and faculty.

## Introduction

### Prevalence of Mental Health Issues

One in five Canadians will experience a mental health issue [1], with those in the age range of 15 to 24 years being the most at risk of meeting criteria for a mood and substance abuse disorder [2]. University students are of particular concern as they have shown significantly higher rates of mental health problems than the general public [3]. In recent years, mental health support services on university campuses have experienced high volumes of appointment requests but are limited in the amount of support they are able to provide. Counseling center directors have previously listed wait-list issues and funding concerns among necessary improvements for Canadian postsecondary counseling services, with 78% reporting being unable to meet the growing demand for services [4]. Today, postsecondary counseling center staff have noted that centers are still in need of additional resources to meet student requests [5]. The average wait time to receive mental health treatment services in Canada is 19.3 weeks [6], and students at some institutions may have to wait up to 6 months for individual treatment [7], meaning many students are left without support.

### Impact on Students

Mental health issues have been shown to negatively impact student academic performance [8,9], with stress, anxiety, and sleep difficulties found to be the top three factors most frequently reported by students [10]. Additionally, diagnosed depression has been associated with decreased academic performance [11] and health problems such as back pain, diabetes, irritable bowel syndrome, and migraine headaches [12]. Unfortunately, when students attempt to manage their mental health issues, they do not always engage in healthy coping mechanisms. A survey of 212 American college students found that only 5% reach out to a professional for stress-related management, with more students instead turning to drinking, smoking, and using illegal drugs [13]. Despairingly, avoidance of seeking professional help for assistance with mental health on university campuses is a worldwide problem [3,14-16]. Reasons for not engaging with professional mental health support included perceiving stress as normal for university or graduate school, fear of judgment, shame, and uncertainty of effectiveness [15,16].

Another notable barrier to seeking mental health aid is stigma [17-19], which may be perpetuated or endorsed by others or internalized by the individual [20]. Stigma associated with mental illness precludes many people from seeking face-to-face counseling [20,21], particularly for those experiencing depressive symptoms, stress, and anxiety [22,23]. Results from a study on help-seeking behaviors and access to health care within a university population showed that 20% of students who did not use support services despite reporting symptoms of anxiety and depressive disorders did so because they were worried about what people would think. Another 20% said that they did not seek help because they thought that others wouldn’t understand their problems [16]. Among Japanese university students, feeling ashamed and worrying about other people’s opinions were listed as barriers to visiting mental health professionals [15].

### Issues With Current Resources

Canadian universities currently provide services and programs to support student mental health, but these services and programs have shortcomings. First, the rise in serious mental health concerns has been associated with an increase in demand for on-campus counseling; yet, university counseling centers have not been able to meet these demands with adequate staffing [5,6]. Furthermore, on-campus counselors are often advised to only take on patients short-term, with policies often limiting the number of sessions allowed per patient [4,24]. Although these policies may allow for a greater number of students to access counselors, it does not necessarily ensure that clients are receiving the long-term support that they are seeking or need. Some institutions have attempted to address this concern by providing group counseling sessions. However, as many students have reported concerns of stigma as a barrier to seeking mental health support [14,25], they may avoid group sessions for fear of being recognized by group members. Additionally, lack of time is a notable barrier to mental health service use [26,27], and group therapy sessions may be too time consuming for students to commit to.

### Mindfulness and Mobile Health (mHealth) Apps as Alternative Resources

Taking into account the barriers to current university mental health support services, an appeal can be made for an alternative, more accessible service. Mobile health (mHealth) phone apps may be a service that addresses these barriers. Mobile phones are integrated into the daily lives of nearly all university community members and can be discreetly used by students to participate in app-based mental health support programs. Individuals seeking help can receive online assistance in a nonthreatening manner that is also feasible and capable of reaching a wide number of people [28]. mHealth apps have high acceptability among users [29,30] and are reportedly more comfortable to use in public compared with other intervention formats such as when used to track diet for weight loss purposes [29]. They are publicly accessible, can be used at a person’s discretion, and hold promise of appealing to people who are not clinically diagnosed with a mental illness but nonetheless have concerns. mHealth apps may also cater to people’s desire to manage their problems on their own [14,28] by providing a support that can be independently used. Additionally, mHealth apps are user friendly: they are easy to use, have minimal time commitment [29], and can be used at any time that is convenient or necessary. These features can help ensure user anonymity and accessibility, making mHealth apps an appealing mental health support alternative.

Apps provide an inconspicuous and convenient mode of delivery for health interventions and may assist in improving accessibility of evidence-based monitoring and self-help [30]. A systematic review of 24 studies concerning the behavioral functionality of mHealth apps found that apps may provide a feasible delivery method for health interventions and have shown potential to bring about behavioral changes such as increasing physical activity and reducing alcohol consumption [29]. However, a systematic review of studies concerning mHealth app efficacy conducted by Donker et al [30] found that of over 3000 mental health apps available at time of study, only 8 studies within the systematic review were identified as evidence-based and only one study utilized a university sample. The latter study evaluated the effectiveness of an Italian mHealth app called Mobile Stress Management at decreasing anxiety and improving coping skills in female university students in comparison to a control condition. This study, as described in the Methods section, advances the literature by including nonfemale participants, using an English mHealth app, and evaluating additional outcomes. Donker et al [30] also found measurements regarding sleep disturbances and anxiety disorders to be particularly lacking in the literature. Similarly, Grist et al [31] argue that there is an insufficient amount of evidence to support the effectiveness of mHealth apps supporting the mental health of adolescents and youth. Both Payne et al [29] and Donker et al [30] identified small sample sizes as a limitation in the current literature on mHealth apps in their systematic reviews. A total of 227 participants were recruited across the 8 studies reviewed by Donker et al, and 17 of the 24 studies reviewed by Payne et al had sample sizes under 100.

Preliminary studies of nonapp-based interventions have shown mindfulness to be a promising tool for helping university students manage their stress and anxiety. Mindfulness is described as the focusing of attention on the present moment, including an awareness of the body and thoughts that is accomplished without judgment [32]. For example, one study involving first-year undergraduate students found that adapted mindfulness-based stress reduction (MBSR) interventions may improve students’ physiological and psychological well-being [33]. The adapted MBSR techniques included reading assignments, discussion, meditation, and yoga and were performed 2 hours a week for 8 weeks. Students demonstrated enhanced personal-emotional adjustment and reduced physiological stress. Similarly, researchers at the University of Northampton found that students who participated in an 8-week mindfulness-based program demonstrated significant decreases in perceived stress, anxiety, and depression as compared with a wait-list control group [34]. These findings were supported by a study in 2014 involving 458 university students that found mindfulness to be associated with improved mental health (eg, reduction in symptoms of depression, anxiety, hostility, and paranoia) [35]. Mindfulness-based interventions are increasing in public interest, and these preliminary findings suggest it may be a promising way of helping university students improve their mental health.

### Current Gap in the Literature

Recent studies have shown app-based supports and mindfulness to be promising ways of addressing mental health concerns, yet, there is a lack of research regarding mindfulness-based techniques delivered through apps. This finding is surprising considering the exhaustive number of mindfulness-based mHealth apps available for purchase. In a recent study evaluating the feasibility of an Internet-based mindfulness training program among university students in Sweden, researchers found that users generally enjoyed the program and its flexibility regarding time and location of use, yet, found no significant intervention effect on psychological well-being or depression symptoms when compared with an Internet-based “expressive writing” program [36]. It is important to note that the mindfulness training was extensive—with participants encouraged to practice 30 to 45 min a day for 6 or 7 days a week. Evaluation of mindfulness-based apps that are less time-intensive than the typical length of in-person mindfulness-based training is needed.

### Research Objective and Hypotheses

This study addressed this gap in the literature by evaluating the efficacy of a commercially available mindfulness-based app, called DeStressify by Stress Refuge Inc (hereafter “DeStressify”), on stress, anxiety, and depressive symptomology within a university population. The cofounder and chairman of Stress Refuge, Inc claimed DeStressify was initially developed for teachers, organizations, and the general public. It was launched in 2014, and a study conducted by DeStressify staff showed a 20% reduction in self-reported stress levels after 4 weeks of app use within a sample of teachers (Ulco Visser, email communication, September 14, 2017). However, no previously published research evaluating DeStressify has been found by the authors of this study.

The app contains a core plan that delivers mindfulness-based exercises through audio, video, or text files that require between 3 to 23 min to complete. Example titles of these exercises include grounding visualization, gratitude, imagining the life you want, and finding meaning. There are free and purchasable “pro” versions of the app available for download. The core plan is available on both the free and pro version of DeStressify, with the pro version offering additional features including “my friends,” “nutrition,” and “shop” options. It was hypothesized that students who use DeStressify would report significantly lower stress, anxiety, and depressive symptomatology as compared with a wait-list control sample post intervention. Secondary outcomes relevant for this specific study population included sleep behavior, work or class absenteeism, work or school productivity, and quality of life (QoL). It was hypothesized that compared with matched wait-list control participants, the participants using DeStressify would report significantly greater sleep quality, school or work productivity and QoL and significantly less class or work absenteeism at postintervention.

## Methods

### Design

In the systematic review of mHealth app studies by Donker et al [30], recruitment within each study ranged from 8 to 117 participants. For this pilot, exploratory study, sample size was calculated through a power analysis using G*Power statistical power analysis software [37]. Effect size estimate calculations were made with the Cohen Perceived Stress Scale (PSS) values from a study by Chang et al [38] in which university students participated in an 8-week MBSR intervention that included group meditation sessions and home practice. Cronbach alpha was set at .05, power at 0.80, and effect size at 0.37. A power analysis indicated a sample size of 61 would be required to test the hypotheses. It is important to note that the intervention used by Chang et al [38] included 20 hours of in-class mindfulness practice and 36 hours of assigned home practice. In consideration of the power analysis, the intervention design differences between this study and that of Chang et al [38], the sample size range in studies reviewed by Donker et al [30], and an anticipated dropout of some participants from pre- to postintervention, a recruitment goal of 200 participants was used.

### Procedure

Participants were recruited through poster advertisements, in-class announcements, and emails to administrative assistants of various faculties across the University of British Columbia (UBC) Okanagan campus. Individuals interested in participating in the study emailed the researcher assistant and received a link to the Web-based eligibility survey, consent form, and baseline survey. Eligibility criteria included (1) enrollment in full course load during the winter term at the UBC Okanagan campus in an undergraduate program, (2) ownership of a smartphone, (3) regular access to the Internet, and (4) fluent comprehension of the English language. Participants indicated consent by clicking an “I consent” button after reading an information page regarding the study. Following the completion of the baseline survey, participants were randomized into either an experimental or wait-list control condition using a computer-generated random numbers table. Random numbers were generated in batches of 50 with equal counts for both treatment conditions (ie, 25 total for each). Individuals in the experimental condition were provided the pro version of DeStressify and were asked to not engage with other features of the app during the course of the study. According to the app’s website, it takes about 1 month to complete the core plan if practicing 3 days a week. To help ensure that participants were completing the core plan, and in recognizing that users may not engage with the app as frequently as recommended, individuals in the experimental condition were instructed to use the app’s core plan 5 days a week for 4 weeks. Participants could set reminders to use the app through the app itself, and an email reminding participants that they were to receive a follow-up email after 4 weeks of app use was sent to participants in the experimental condition half way through the intervention. Individuals in the control condition were given no treatment and no intervention material until after the postintervention survey was completed, at which time they were provided the app and similar guidelines for use as the experimental condition. The follow-up period was 4 weeks post baseline, at which point all participants were sent a second Web-based questionnaire. All participants who completed both surveys received an electronic Can $25 Amazon gift card. Data were collected and stored on secure systems and accessed through computers with password protection and encryption. This study was approved by the institutional review board.

### Measures

All participants completed a baseline survey composed of questions regarding demographic characteristics and 6 validated self-reported measures of stress, anxiety, depressive symptomatology, sleep quality, QoL, and work productivity. Each measure was presented on a separate page in the survey, and participants were able to use a “back” button to review and change their answers before submitting their completed surveys. Participant responses were identified by their email addresses.

Demographic measurements of sex, age, income, ethnic origin, educational background, and university program of enrollment were included at the baseline assessment. Participants were also asked to identify any mental health disorder diagnoses they had, whether they were using mental health services, and if so, for how long.

Perceived stress was measured using the PSS, which contained 10 items requiring respondents to indicate how often they felt or thought a certain way over the past month [39]. Scores could range from 0 to 40 with a mean score of 14.2 for people in the age range of 18 to 29 years and 12.1 and 13.7 for males and females, respectively [39]. The PSS has shown validity and reliability within samples of college students [40].

Anxiety was measured using the State-Trait Anxiety Inventory for adults, which contained 40 items in two subscales: state anxiety and trait anxiety [41]. Each subscale included 20 statements that people may use to describe how they feel. Participants were asked to indicate how accurately each statement described them presently for state anxiety and in general for trait anxiety. Responses were scored to yield a collective score that could vary between 20 and 80 for each subscale. Both subscales have shown reliability, validity, and internal consistency within samples of high school and college students [41].

Symptoms of depression were measured using The Quick Inventory of Depressive Symptomatology Self-Report (QIDS-SR), with ratings made in consideration of the past 7 days [42]. It contained 16 items that divided into the nine symptom criterion domains associated with the Diagnostic and Statistical Manual of Mental Disorders, 4^th^ Edition for major depressive disorder (MDD): low mood, concentration, self-criticism, suicidal ideation, loss of interest in activities, energy or fatigue, sleep disturbance, changes in appetite or weight, and psychomotor agitation or retardation. Scores, which can range from 0 to 27, can be divided into 5 categories associated with different classifications of depression severity: none, mild, moderate, severe, and very severe, with a five-point change in score associated with a change in classification [43]. The QIDS-SR has shown validity and reliability within a sample of adults with chronic, nonpsychotic MDD [42].

Sleep quality was measured using the Pittsburg Sleep Quality Index (PSQI), which asked participants to complete the measure in consideration of their usual sleep habits over the past month [44]. The measure contained 19 questions for respondents that were scored and combined to form 7 component scores: subjective sleep quality, sleep latency, sleep duration, habitual sleep efficiency, sleep disturbances, use of sleeping medication, and daytime dysfunction. These component scores were added to form a global PSQI score. A global score greater than 5 suggests that the respondent may have difficulties in 2 or more components [44]. An additional 5 questions were included for respondents with bed partners or roommates, although these questions did not contribute to score calculations. The PSQI has shown reliability and validity among “good” and “poor” sleepers with and without sleep-related disorders [44].

Health-related QoL was measured using the RAND 36-Item Health Survey, which included 36 items to address 8 health concepts: physical functioning (eg, ability to perform any physical activity such as bathing and eating), bodily pain, physical health problems that limit ability to perform a specific role (eg, work and daily activities), personal or emotional problems that limit ability to perform a specific role, emotional well-being, social functioning, energy or fatigue, and general health perceptions (ie, beliefs regarding overall health) [45,46].Responses to all items were scored out of 100, and a score for each of the 8 health concepts was calculated by averaging a collection of item scores. These scores represent percentages, where “a higher score defines a more favorable health state” [47]. The RAND 36-Item Health Survey is a popular measure of QoL and has shown acceptable levels of reliability, validity, and internal consistency [48].

Work productivity was measured using the Work Productivity and Activity Impairment Questionnaire: General Health V2.0 (WPAI) [49]. Respondents completed 2 to 6 items in consideration of “the effect of (their) health problems on (their) ability to work and perform regular activities” [49], where health problems were defined as “any physical or emotional problem or symptom” [49]. Responses were scored and 4 subscales, expressed in percentages, were calculated: absenteeism, “presenteeism” [49], work productivity loss, and activity impairment. Higher scores indicated greater impairment and productivity loss. The WPAI has shown reliability and validity within a sample of working individuals [49].

Two additional questions were included in the follow-up surveys for both the experimental and control conditions. For these questions, participants identified whether they believed their sleeping and work or school productivity improved, worsened, or stayed the same since baseline measurements were taken.

**Figure 1:**
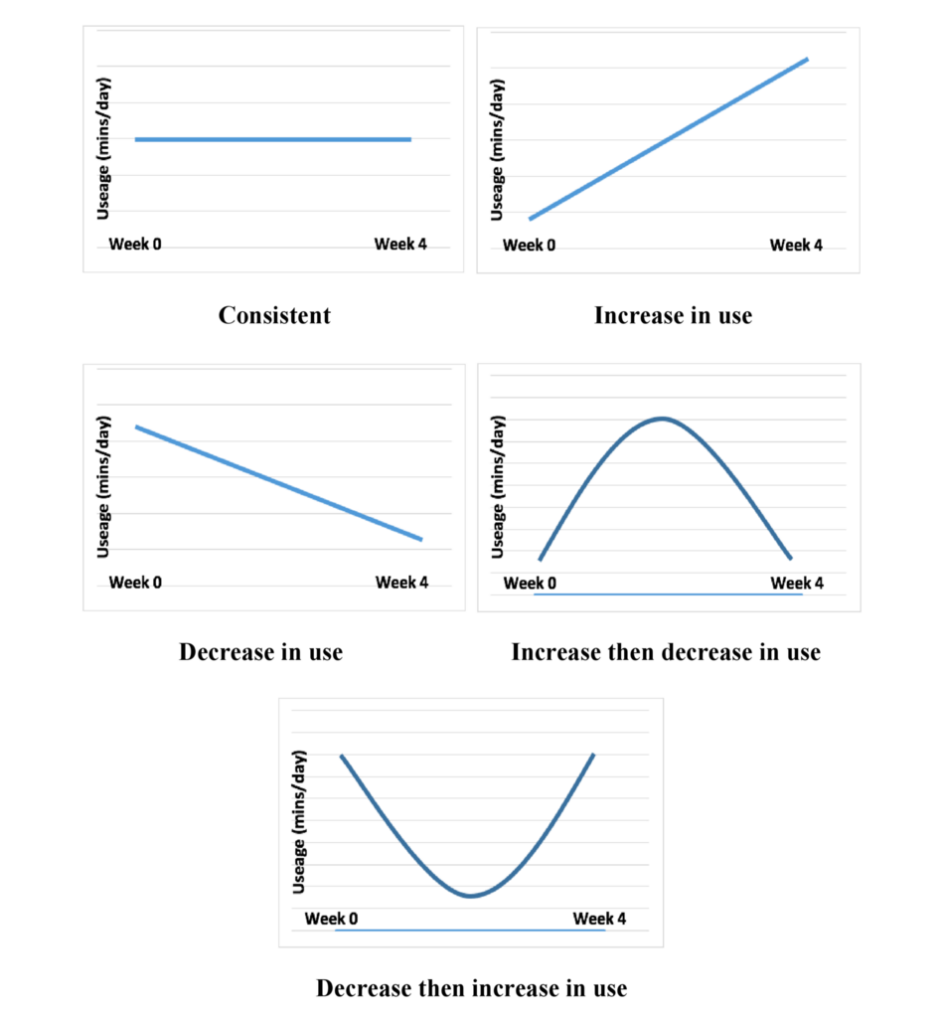
Graphic representations of app use trends provided in experimental condition postintervention survey.

App use questions were included in the follow-up survey for the experimental condition. Participants were asked how frequently they used the app in comparison with what was requested at the beginning of the study and their pattern of app use over the 4 weeks. App use frequency was measured using a 10-point scale ranging from “did not use at all” to “used as often as requested.” Response options to describe patterns of app use were increased, increased then decreased, consistent, decreased then increased, and decreased and included graphic representations (see Figure 1). All app use data were self-reported.

### Analytic Plan

Data were analyzed using Statistical Package for the Social Sciences (SPSS) version 23 (IBM Corp). Age distribution of treatment conditions were compared using the Mann Whitney *U* test. Distribution of sex, mental health disorder diagnoses, and mental health service use within treatment conditions were compared using chi-square tests. Distribution of program enrollment within treatment conditions was compared using Fisher exact test. Ethnicity distributions were compared using either chi-square test or Fisher exact test, depending on whether or not the assumptions of the chi-square test were met within each ethnicity category. Changes in pre- and postintervention scores between treatment conditions for measurements of stress, depression, state and trait anxiety, sleep quality, QoL subscales, and work productivity subscales were assessed using analysis of covariance (ANCOVA). For all ANCOVA, postscores were treated as the dependent variable and prescores the covariate. A multivariate analysis of covariance (MANCOVA) was also conducted in which the baseline scores for both state and trait anxiety were assigned as covariates, and the dependent variables were the postintervention state and trait anxiety scores. Chi-square test was conducted to identify differences in perceived work productivity. An alpha level of .05 was used in all statistical tests of significance, and effect size was determined using partial eta squared values. In alignment with suggestions by Cohen, partial eta squared values of .0099, .0588, and .1379 were used to correspond to small, medium, and large effect sizes, respectively [50]. Normality was tested using the Kolmogorov-Smirnov test. Raw scores that were not normally distributed were transformed through square root calculations to produce normality [51]. Univariate outliers were identified as having z-scores with magnitude greater than 3.29 (*P*<.001). Mutivariate outliers were identified as having Mahalanobis distance values greater than χ^2^_2_=13.8 when analyzing anxiety scores and χ^2^_8_=26.1 when analyzing QoL scores, *P*<.001 [51]. When outliers were present, analyses were run with and without outliers.

## Results

### Demographics

**Figure 2:**
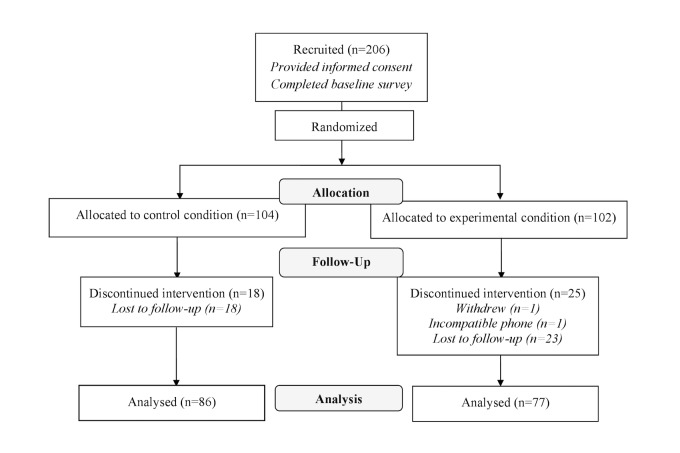
Consolidated Standards of Reporting Trials (CONSORT) flow diagram (template obtained from consort-statement.org).

Responses from 206 students at UBC Okanagan were collected at baseline, with 104 randomized into the control condition and 102 into the experimental condition (see Figure 2). Of the 206 participants, 43 were excluded from analysis because of failure to complete postsurveys (n=41), having a phone that did not support the app (n=1), or a family emergency (n=1). This resulted in 163 responses being used in analysis (86 control, 77 experimental). There were no differences in age, sex, ethnicity, program enrollment, mental health diagnosis percentage, and mental health service use between conditions, *P*>.05 (see Table 1). The percent of participants in both conditions self-reporting mental health diagnoses is noteworthy, although a chi-square test confirmed that the number of people reporting such diagnoses was not statistically different between conditions, *P*=.18. There was also no difference in the percentage of participants in each condition who utilized health care services, *P*=.80. Human kinetics, general arts and sciences, and nursing were the three most common programs for enrollment in both treatment conditions.

**Table 1:** Demographics of treatment conditions.

Characteristic		Control (n=86)	Experimental (n=77)
**Age (years)**			
	Range	16-47	18-27
	Average	20.9	20.3
**Sex, n (%)**			
	Female	58 (67)	45 (58)
**Ethnicity (3 most predominant listed), n (%)**			
	White	61 (71)	50 (65)
	Chinese	9 (11)	12 (16)
	South Asian	5 (6)	9 (12)
**Program enrollment, n (%)**			
	Human kinetics	22 (26)	12 (16)
	First year arts and sciences	12 (14)	14 (18)
	Nursing	11(13)	11 (14)
**Mental health diagnosis^a^**			
	Yes, n (%)	12 (14)	17 (22)
	Bipolar, (n)	1	0
	Depression, (n)	4	5
	Anxiety, (n)	4	6
	Other, (n)	3 (obsessive-compulsive disorder and dermotilamania; depression, anxiety, and mania; attention-deficit hyperactivity disorder [ADHD])	11 (adjustment disorder, anxiety, and depression; 3 ADHD; attention-deficit disorder; depression, anxiety, and ADHD; post-traumatic stress disorder [PTSD]; binge eating; depression and anxiety; bipolar, depression, and anxiety; depression, anxiety, and PTSD)
**Mental health service use, n (%)**			
	Yes	10 (12)	10 (13)

^a^Diagnoses taken verbatim from participant responses.

### Stress

Perceived stress scores within the experimental condition decreased in value, whereas control condition scores slightly increased in value from baseline to postintervention (see Table 2). However, differences in scores between conditions at postintervention did not reach statistical significance, *F*_1,160_=3.54, *P*=.06, η_p_^2^=.02. Reliability of the PSS and other validated surveys, as determined by Cronbach alpha, is provided in Table 3.

### Depression

One participant was excluded from analysis of the QIDS-SR questionnaire as baseline responses were not provided for this measure by the participant. Raw scores were transformed for normality. Mean values for the QIDS-SR scores in Table 2 were calculated using raw data. Postintervention transformed QIDS-SR scores for the experimental condition showed no significant difference from the control condition, *F*_1,159_=3.01, *P*=.09, η_p_^2^=.02, when controlling for baseline scores. Tests were reconducted using nontransformed data, and results were similar, *F*_1,159_=3.54, *P*=.06, η_p_^2^=.02.

### Anxiety

One multivariate outlier was identified in the control condition. No univariate outliers were observed in either trait or state anxiety scores. Overall, omnibus *F* tests of the MANCOVA were significant when the outlier was included, *F*_1,160_=4.25, *P*=.02, η_p_^2^=.05, and excluded, *F*_1,159_=4.13, *P*=.02, η_p_^2^=.05. ANCOVA results demonstrate that individuals in the experimental condition reported less trait anxiety, *F*_1,160_=8.23, *P=*.01, η_p_^2^=.049, than individuals in the control condition after 4 weeks of using the DeStressify app. State anxiety scores did not significantly differ between conditions at postintervention, *F*_1,160_=1.93, *P*=.17, η_p_^2^=.01.

**Table 2:** Mean values of measures for control (n=86) and experimental (n=77) treatment conditions at baseline and 4 weeks post intervention, excluding outliers. Standard deviations are included in parentheses.

Dependent variable	Pre		Post	
	Control Mean (SD^a^)	Experimental Mean (SD)	Control Mean (SD)	Experimental Mean (SD)
State-Trait Anxiety Inventory (STAI) state^b^	44.7 (13.0)	43.0 (12.0)	43.4 (13.2)	40.1 (12.1)
STAI trait^c^	47.6 (11.1)	47.4 (10.6)	47.5 (10.8)	44.5 (9.4)
QIDS-SR^d^	8.1 (4.5)	8.4 (4.3)	7.4 (4.7)	6.4 (3.9)
Physfunct^e,f^	93.8 (7.5)	92.1 (9.8)	93.3 (10.1)	92.1 (10.6)
Physlim^e^	78.2 (32.3)	79.2 (33.3)	77.0 (33.7)	83.8 (30.6)
Emolim^e^	50.4 (42.4)	52.4 (40.3)	49.2 (41.8)	58.4 (42.6)
Energy^e^	45.1 (17.8)	46.2 (20.1)	41.1 (18.9)	48.9 (19.4)
Emowell^e^	60.9 (18.6)	61.1 (20.0)	58.7 (20.1)	66.0 (17.2)
Socialfunct^e^	72.2 (22.4)	74.2 (24.4)	71.4 (24.5)	77.0 (20.2)
Pain^e,g^	78.3 (19.2)	83.5 (19.0)	78.0 (18.6)	84.3 (18.0)
Genhealth^e^	64.7 (20.5)	63.3 (19.5)	61.7 (20.4)	67.5 (17.4)
PSS^h^	19.6 (7.7)	18.6 (6.8)	19.8 (6.7)	17.8 (6.2)
WPAI^i^ missedtime^j,k^	4.2 (13.6)	2.1 (3.5)	0.1 (0.2)	0.1 (0.1)
WPAI impairedtime^j,k^	16.6 (21.9)	11.0 (21.7)	20.7 (26.0)	14.3 (23.6)
WPAI overallworkimpair^j,k^	18.1 (25.4)	11.6 (23.4)	28.1 (32.1)	16.6 (27.4)
WPAI activimpair^j^	25.7 (26.5)	22.9 (25.0)	24.0 (26.7)	18.1 (23.5)
PSQI^l^	7.0 (2.8)	6.9 (3.1)	7.0 (3.7)	6.2 (3.1)

^a^SD: standard deviation.

^b^STAI state: State-Trait Anxiety Inventory for Adults—state anxiety.

^c^STAI trait: State-Trait Anxiety Inventory for Adults—trait anxiety.

^d^QIDS-SR: Quick Inventory of Depressive Symptomatology Self-Report.

^e^Physfunct, Physlim, Emolim, Energy, Emowell, Socialfunct, Pain, Genhealth: RAND 36-Item Health Survey—Physical functioning subscale, role limitations because of physical health subscale, role limitations because of emotional health subscale, energy or fatigue subscale, emotional well-being subscale, social functioning subscale, pain subscale, and general health subscale.

^f^Physfunct scores were calculated using n=156 (n_exp_=75, n_con_=81).

^g^Pain scores were calculated using n=162 (n_exp_=76, n_con_=86).

^h^PSS: Perceived Stress Scale.

^i^WPAI: Work Productivity and Activity Impairment Questionnaire: General Health V2.0.

^j^WPAI missedtime, impairedtime, overallworkimpair, activimpair: Percent work missed because of health, percent impairment while working because of health, percent overall work impairment because of health, and percent activity impairment because of health.

^k^WPAI impairedtime and overallworkimpair scores were calculated using n=50 (n_exp_=21, n_con_=29).

^l^PSQI: Pittsburg Sleep Quality Index.

**Table 3:** Cronbach alpha values for all validated measures using postintervention data (n_con_=86, n_exp_=77).

Survey component	Cronbach alpha
PSS^a^	.86
QIDS-SR^b^	.80
State-Trait Anxiety Inventory (STAI) state	.95
STAI trait	.90
PSQI^c^	.72
Physfunct	.88
Physlim	.83
Emolim	.83
Energy	.77
Emowell	.82
Socialfunct	.83
Pain	.83
Genhealth	.78

^a^PSS: Perceived Stress Scale.

^b^QID-SR: Quick Inventory of Depressive Symptomatology Self-Report.

^c^PSQI: Pittsburg Sleep Quality Index.

### Sleep Quality

In regards to the baseline scores, there was 1 outlier from each treatment condition that was removed from analysis for sleep quality. Raw scores were transformed for normality. Transformed values were more normally distributed, although both the raw and the transformed scores were significant when tested for normality. Nonetheless, analysis of variances (ANOVAs) were conducted as they are robust given the data’s traits [51]. There was no significant differences between treatment conditions in the postintervention scores for both raw, *F*_1,158_=2.51, *P*=.12, η_p_^2^=.02, and transformed scores, *F*_1,158_=1.89, *P*=.17, η_p_^2^=.01, when outliers were excluded. Results were similar when outliers were included (raw: *F*_1,160_=2.58, *P*=.11, η_p_^2^=.016; transformed: *F*_1,160_=1.91, *P*=.17, η_p_^2^=.01).

### Quality of Life

Distributions of all subscores for the RAND 36-Item Health Survey were non-normally distributed with the exception of energy or fatigue at baseline for the experimental condition, energy or fatigue at follow-up for both treatment conditions, and general health at follow-up for the control condition. Additionally, the assumption of homogeneity of covariance matrices was not met. However, ANOVA is robust to violations of normality for this data when outliers are excluded [51], and MANOVAs are robust to heterogeneity of covariance matrices when sample sizes are equal [50,51].

Researchers identified one univariate outlier from the pain subscale, seven univariate outliers from the physical functioning subscale, and six multivariate outliers. MANCOVA was conducted with outliers, and a significant interaction effect was found between condition assignment and time, *F*_1,160_=2.06, *P*=.04, η_p_^2^=.10, warranting examination of individual QoL subscales, as discussed below. Eight outliers were removed from analysis for a second MANCOVA, resulting in the test being conducted on a sample size of 81 control condition participants and 74 experimental condition participants. Trends were similar in that mean subscale values decreased within the control condition from baseline to postintervention and increased within the experimental condition (see Table 2), although these trends were not found to be significant, *F*_1,152_=1.86, *P*=.07, η_p_^2^=.10.

Vincent [52] warns that significance of certain dependent variables may be masked by nonsignificant variables in MANOVAs and therefore recommends ANOVA with the Bonferroni adjustment for assessing specific variables of interest. Thus, ANCOVAs were conducted on all subscales of the RAND 36-Item Health Survey. The general health subscale was shown to significantly differ in postintervention scores between treatment conditions, *F*_1,160_=12.44, *P*=.001, η_p_^2^=.07, such that scores decreased in the control condition and increased in the experimental condition as illustrated in Table 2. A significant difference was also found between treatment conditions in regards to postintervention energy or fatigue subscale scores, *F*_1,160_=8.19, *P*=.01, η_p_^2^=.05, with similar trends as the general health subscale. The results of the emotional well-being subscale did not meet the assumption of homogeneity of regression slopes for ANCOVA and were therefore analyzed using repeated measures ANOVA.

**Table 4:** Participant count for responses regarding changes in perceived work or school productivity over 4 weeks between baseline and postintervention measurements. Expected count is provided in parentheses.

Treatment	Control	Experimental
*I think I was MORE productive*	14 (22.2)	28 (19.8)
*I think I was LESS productive*	32 (26.4)	18 (23.6)
*I think my productivity stayed about the same*	40 (37.5)	31 (33.5)

**Figure 3:**
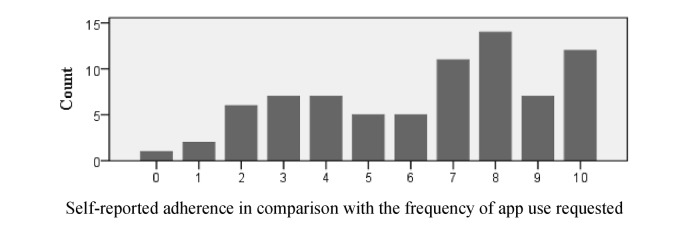
Response counts from participants in the experimental condition when asked to identify how often they used the app in comparison with the frequency of use requested.

There was no main effect of time, *F*_1,161_=1.21, *P*=.27, η_p_^2^=.01, or condition assignment *F*_1,161_=1.89, *P*=.17, η_p_^2^=.01; however, there was an interaction effect, *F*_1,161_=8.13, *P*=.01, η_p_^2^=.05, such that scores decreased over time for the control condition and increased over time for the experimental condition (see Table 2). All other tests were insignificant.

### Work Productivity

Of the 163 participants who completed the follow-up survey, 29 people from the control condition and 21 from the experimental condition (n=50) reported having work at both baseline and postintervention and therefore completed all components of the WPAI. A subscore was calculated using these work-related data, labeled “percent overall work impairment due to health” [49]. No significant difference was found between treatment conditions, *F*_1,47_=1.10, *P*=.30, η_p_^2^=.02.

All 163 participants completed the question, “During the past seven days, how much did your health problems affect your ability to do your regular daily activities, other than work at a job?” [49] This question was used to calculate percent activity impairment because of health. No significant difference was found between treatment conditions, *F*_1,160_=1.72, *P*=.19, η_p_^2^=.01.

When participants were asked to choose a description that best described how their work or school productivity has changed over the past 4 weeks, there was an association between treatment condition and responses, *P*=.01. More participants than expected by chance in the experimental condition reported an improvement in their productivity. Conversely, fewer participants than expected by chance in the control condition reported improved productivity. These results (Table 4) suggest that those in the experimental condition reported being more productive than those in the control condition.

### Patterns of App Use and Self-Reported Adherence

One participant did not report app use trends and was thus excluded from the data regarding response frequencies of participants in the experimental condition (n=76) for self-reported trends in app use. The most frequently reported patterns of app use were consistent use (n=23) and decrease in use (n=23). The most infrequently reported app use trend was increase in use (n=4). The remaining responses of increase then decrease in use and decrease then increase in use had 14 and 11 responses, respectively.

When adherence was self-reported using a scale from 0 to 10 by participants in the experimental condition, the mean rating was 6.36 (standard deviation 2.79) with a median of 7. The most frequently reported adherence rating was 8. Counts for each response option are provided in Figure 3.

## Discussion

### Principal Findings

It was hypothesized that students who use DeStressify would report significantly lower stress, anxiety, depressive symptomatology, and class or work absenteeism and significantly greater sleep quality, school or work productivity, and QoL as compared with a wait-list control sample post intervention. The results support hypotheses that short-term use of DeStressify can reduce trait anxiety and improve general health, energy, emotional well-being, and work or school productivity in university students but do not support the other hypotheses. These findings are somewhat consistent with related literature, which have shown that MBSR techniques that are delivered in person and through one- to two-hour-long sessions improve physiological and psychological well-being in university students [33-35]. Inconsistencies may be attributed to the difference in intervention delivery methods (ie, through a mobile phone rather than in person), shorter sessions, and lack of experimental control over app use frequency. Considering the study’s design, these findings are encouraging as universities are in need of accessible alternative mental health management tools and services for students but should be considered cautiously given the small effect sizes.

The sample population of this study is representative of a large number of university students in Canada. Many universities are primarily composed of full-time undergraduate students, with a greater proportion of females attending in comparison with males. Specifically, 95% of first-year undergraduate students at Canadian universities are attending full-time, with 66% being female [53]; participants in this study were of similar demographics (enrolled full-time, 63.1% [103/163] female). Additionally, UBC is a Western Canadian university with approximately 8000 students registered in undergraduate programs on the Okanagan campus [54]. This size is comparative to small- to medium-sized Canadian universities. 

The participant dropout rate from pre- to postintervention was 19.9% (41/206). This rate is comparable with the dropout rates of other studies using Web-based programs to support mental health [55]. One healthy lifestyle Web-based intervention with an adult population had an attrition rate over 75% at 1 month post intervention [56]. Apps, in particular, lose over 75% of daily active users 3 days after download [57]. The majority of dropouts were considered lost to follow-up. Two participants were an exception to this: one participant dropped out at the beginning of the study when they identified that the app did not work on their mobile device. A second participant identified their desire to drop out for personal reasons after they were invited to complete the follow-up survey.

Previous studies have found that students reportedly avoid mental health services such as counseling and medication because of fear of stigmatization, lack of time, and cost. Apps are an appealing platform for mental health support for university students that avoid many of the barriers associated with other forms of support, including those aforementioned and until recently have lacked evidence regarding effectiveness. App-based supports such as DeStressify can help university students avoid the stigma associated with mental health, as a large majority of students possess mobile phones and frequently engage with them, making app use a more discrete form of mental health maintenance. Additionally, mHealth apps do not generally require a large amount of time to use; the practices provided in DeStressify are approximately 10 min in length—much shorter than a standard counseling session. Participants in the experimental condition of this study were instructed to use the app 5 days a week, with no specifications regarding when it should be used. This allowed for greater flexibility in scheduling and thus, greater convenience, whereas still resulting in changes to trait anxiety, certain QoL components, and work or school productivity. In addition, apps are often inexpensive. The DeStressify app that was provided to participants is publicly available for Can $8.49 at the Apple iTunes store [58] and Can $8.23 at the Google Play store [59].

The changes in stress, anxiety, and related traits after short-term use of DeStressify are encouraging, yet, the effects of long-term use remain unknown. The most frequently reported patterns of app use for participants in the experimental condition were “consistent” and “decrease in use.” Additionally, the average self-reported adherence rate in comparison to the requested amount was 64% (6.4/10), with 80% (8/10) being the most commonly reported adherence rate. Considering these self-reported adherence patterns, rates, and the magnitude of the changes in measured traits among DeStressify users, it would be interesting to determine whether the improvements observed in the experimental group of this study would persist with prolonged use of DeStressify.

### Limitations

This study is among the first to provide empirical evidence regarding the effectiveness of a mindfulness-based mHealth app on stress, anxiety, depression, and related symptomatology within university students. In recognizing the novelty of this study, areas for future development should also be addressed. Although this study’s objectives did not necessitate the use of a mindfulness measure, future studies should include one as it would provide a greater understanding in to the mechanism of action of DeStressify and would be useful in the design of future mental health apps and support services. Obtaining data directly from the app regarding participant use would also be more accurate than obtaining data from self-reported measures and should be considered in future studies. As some participants provided feedback regarding user satisfaction, future studies may also wish to include a measure of user satisfaction to enrich discussion regarding a mental health app’s effectiveness and acceptance within a university population. Rickard et al [60] recommend providing opportunities for feedback directly in the app, particularly using established measures to allow for comparison between apps. Additionally, participant recruitment was dependent on self-selection, and thus, may not represent a random sample of the university population. However, individuals who would be inclined to use a mental health app may also be more likely to respond to this study’s call for participants.

There is also the possibility that some participants in the control condition downloaded DeStressify, as it is a commercially available app. Therefore, we cannot discount the possibility that scores within the control condition may have been altered because of app use. If this were to have occurred, it is suspected that control condition scores would have been closer to experimental condition scores than what they would have been if the app had not been used.

What’s more, some participants may have previously received mindfulness training and possibly interacted with DeStressify differently than participants who had not previously received mindfulness training. However, it is unknown how previous training would impact results. For example, if participants found the app’s exercises to be similar to their current mindfulness exercises, then they may have incorporated the app more easily into their daily schedule and used it more consistently. This consistent use could have yielded greater changes in their scores. Conversely, the similarities in the exercises could have yielded smaller changes in their scores. Including a measure of previous mindfulness training would be beneficial in future studies to control for its possible effects.

Finally, future directions may include comparing the effectiveness of a mental health app on different subpopulations such as university staff or high school students and gathering data beyond 4 weeks of app use to better understand long-term effectiveness of the app. Additional apps may also be considered, so as to provide a more generalized understanding of mindfulness-based mHealth apps.

### Conclusions

Universities and other similar institutions may benefit from supporting the use of DeStressify or other mindfulness-based mHealth apps. It is a resource that can be easily incorporated into support services and used in addition to other mental health support services. Mindfulness-based mHealth apps such as DeStressify may be of interest to university students who are comfortable with apps and seek to manage their anxiety and mental health through an accessible, inexpensive, and discrete manner. Students interested in methods of anxiety management or mindfulness-based self-driven health support may be encouraged to try using the DeStressify app. As app use is self-directed, institutions that provide students with DeStressify may choose to conduct their own follow-up with students so as to track mental health progress. Regardless, an effective mHealth app would provide another means of addressing stress, anxiety, and related mental health concerns, allowing more students to receive the help they are seeking. This study has demonstrated how DeStressify can assist in improving some of these mental health traits in a short time frame and therefore, may be of interest to universities aiming to diversify their student mental health supports.

## Abbreviations

ANOVA: analysis of variance
ANCOVA: analysis of covariance
MANCOVA: multivariate analysis of covariance
MBSR: mindfulness-based stress reduction
MDD: major depressive disorder
mHealth: mobile health
PSQI: Pittsburg Sleep Quality Index
PSS: Perceived Stress Scale
QIDS-SR: Quick Inventory of Depressive Symptomatology Self-Report
QoL: quality of life
STAI: State-Trait Anxiety Inventory for Adults
WPAI: Work Productivity and Activity Impairment Questionnaire: General Health V2.0
